# Quantitative image analysis applied to revise the taxonomy of the Palearctic *Earophila badiata* species group (Lepidoptera: Geometridae: Larentiinae)

**DOI:** 10.7717/peerj.20620

**Published:** 2026-02-19

**Authors:** Mikael Englund, George Hancock, Elina Laiho, Johanna Mappes, Pasi Sihvonen, Max Söderholm, Alpo Turunen, Kyung Min Lee

**Affiliations:** 1 Zoology Unit, Finnish Museum of Natural History, University of Helsinki, Helsinki, Finland; 2 Centre for Ecology & Conservation, University of Exeter, Penryn, United Kingdom; 3 Organismal and Evolutionary Biology Research Program, Faculty of Biological and Environmental Sciences, University of Helsinki, Helsinki, Finland; 4 Biodiversity Information Facility, Finnish Museum of Natural History, University of Helsinki, Helsinki, Finland

**Keywords:** Integrative taxonomy, Geometrid moths, DNA barcoding, Morphological variation, Species delimitation, Micro-CT imaging, Quantitative image analysis

## Abstract

The geometrid moth *Earophila badiata* (Denis & Schiffermüller, 1775), which occurs widely in the Palearctic realm, has rapidly filled a large gap in its range across southern Finland during the past two decades, prompting a re-evaluation of its taxonomy. Using an integrative taxonomic approach including a quantitative wing image analysis combined with genitalia morphology and mitochondrial DNA barcoding (mtCOI) analyses, we reassessed the status of the described taxa within the *E. badiata* species group. Quantitative analysis of forewing colours revealed strong sexual dimorphism and significant effects of specimen wear and age on colouration, but no consistent morphological differences between the nominotypical subspecies *E. badiata badiata* and taxon *E. badiata fennokarelica* (Kaisila, 1945). Comparative genitalia morphology, including micro-CT imaging, showed no diagnostic differences among closely related *E. badiata*, *E. kolomietsi* Vasilenko, 2003, and *E. pseudobadiata* Vasilenko, 2007, supporting the synonymy of these taxa. Molecular phylogeny and haplotype analysis confirmed monophyly among Eurasian samples with low genetic divergence (<0.63%), but implying a distinct lineage for North African *E. badiata tellensis* (Herbulot, 1957). Based on these findings, we propose synonymizing *E. kolomietsi* and *E. pseudobadiata* with *E. badiata* syn. n. and classify the *E. badiata* taxon *fennokarelica* as a morphological form of *E. badiata* below the subspecific rank. Our results challenge the current subspecies delineation and support a revision of taxonomic boundaries within this group, highlighting the value of integrative taxonomy for resolving complex relationships among closely related species.

## Introduction

**Figure 1:**
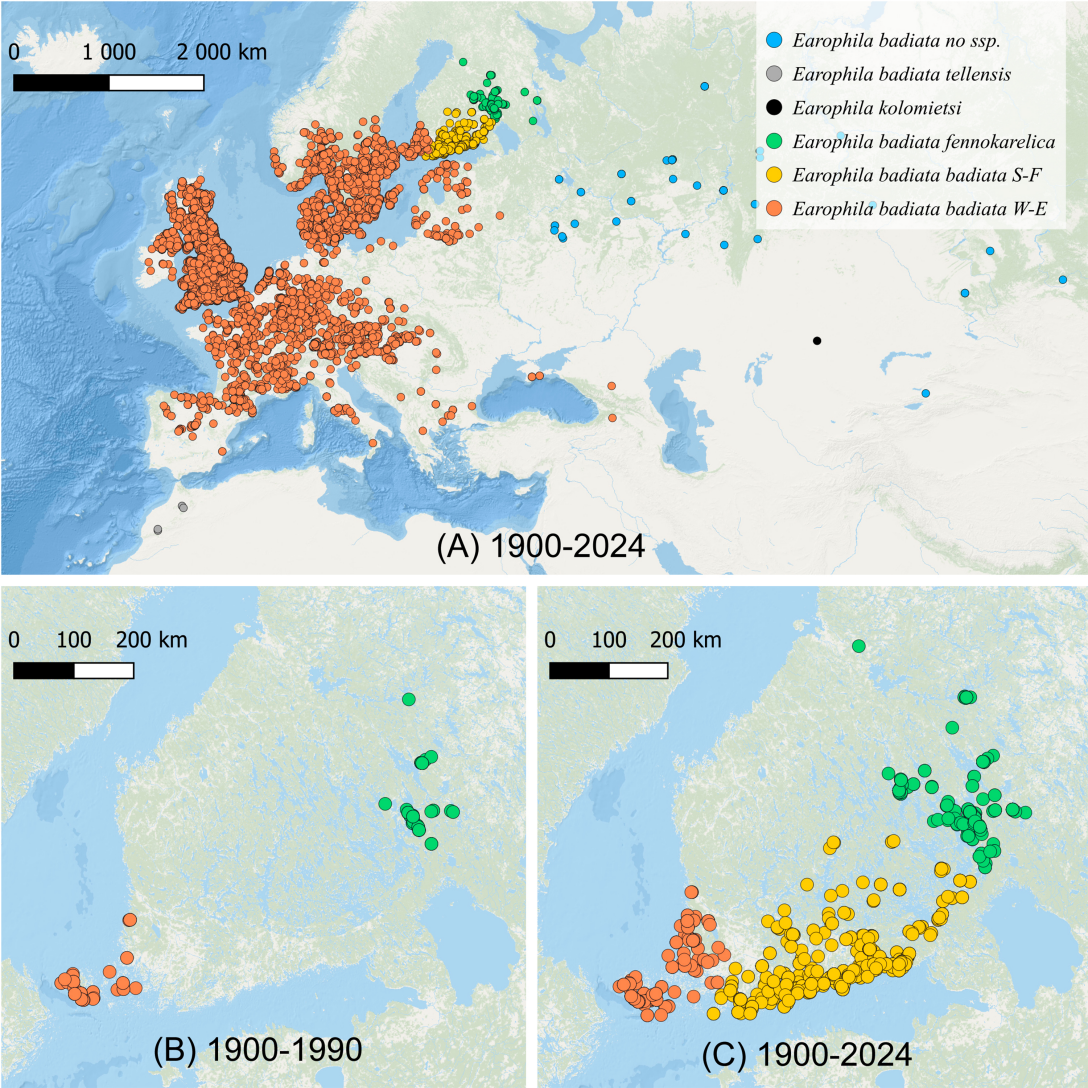
The distribution of Earophila badiata. (A) The distribution in northwestern Palearctic (GBIF, 2024); (B) the distribution in Finland 1900–1990 (FinBIF, 2025), and (C) 1900–2024 (FinBIF, 2025). We use the following colour coding for the *a priori* subspecific annotation of the specimens for all our datasets, figures and tables throughout this study: dark orange for *E. badiata badiata* from Mid and Western Europe including South-West Finland (W-E), light orange for *E. badiata badiata* specimens colonizing South Finland (S-F) from 1990 on, green for *E. badiata fennokarelica* from Finnish and Russian Karelia, blue for *E. badiata* of Russian and Asian origin, grey for *E. badiata tellensis* in North Africa, and black for *E. kolomietsi* (molecular dataset only).

The geometrid moth *E. badiata* has rapidly colonized Southern and Southeastern Finland during the past two decades filling the large gap between two long documented (FinBIF, 2025; Mikkola, Jalas & Peltonen, 1985; Hausmann & Viidalepp, 2012) separate populations of this species in Southwestern Finland and Northern Karelia in Eastern Finland (Figs. 1B, 1C). Whether these two populations were truly allopatric or merely peripatric representing the outskirts of the European population remains uncertain since the distribution pattern is not adequately studied in Northwestern Russia between the continental Europe and North Karelia in Finland.

*Earophila badiata* has a wide Palearctic distribution from North Africa through Europe to Asia (Fig. 1A). Two subspecific taxa, *E. badiata fennokarelica* from Eastern Fennoscandia and *E. badiata tellensis* from the Atlas Mountains have been described, while the nominotypical subspecies distributed in Western and Central Europe is referred as *E. badiata badiata*. Jouko Kaisila described the taxon *E. badiata fennokarelica* as forma geographica (f. geogr.) based on several specimens collected in 1943–1944 from the Russian Eastern Karelia (Kaisila, 1945), and the taxon is later considered to be of subspecific rank according to ICZN Code of Nomenclature (ICZN, 1999, Ch.1 Art. 45.6.4.). According to the original description, *E. badiata fennokarelica* differs from the nominate form by its paler and less contrasting forewing coloration, and smaller size. This taxon was also found at the same time in the Finnish province of North Karelia. In more recent taxonomic work (Hausmann & Viidalepp, 2012; Scoble, 1999) the specimens from the Karelian population together with those from European part of Russia were considered to belong to the subspecies *E. badiata fennokarelica.* Sergei Vasilenko described two new species in the species group, *E. kolomietsi* and *E. pseudobadiata* from central and eastern parts of northern Asia distinguishable from *E. badiata* only based on genitalia morphology (Vasilenko, 2003; Vasilenko, 2007).

**Figure 2:**
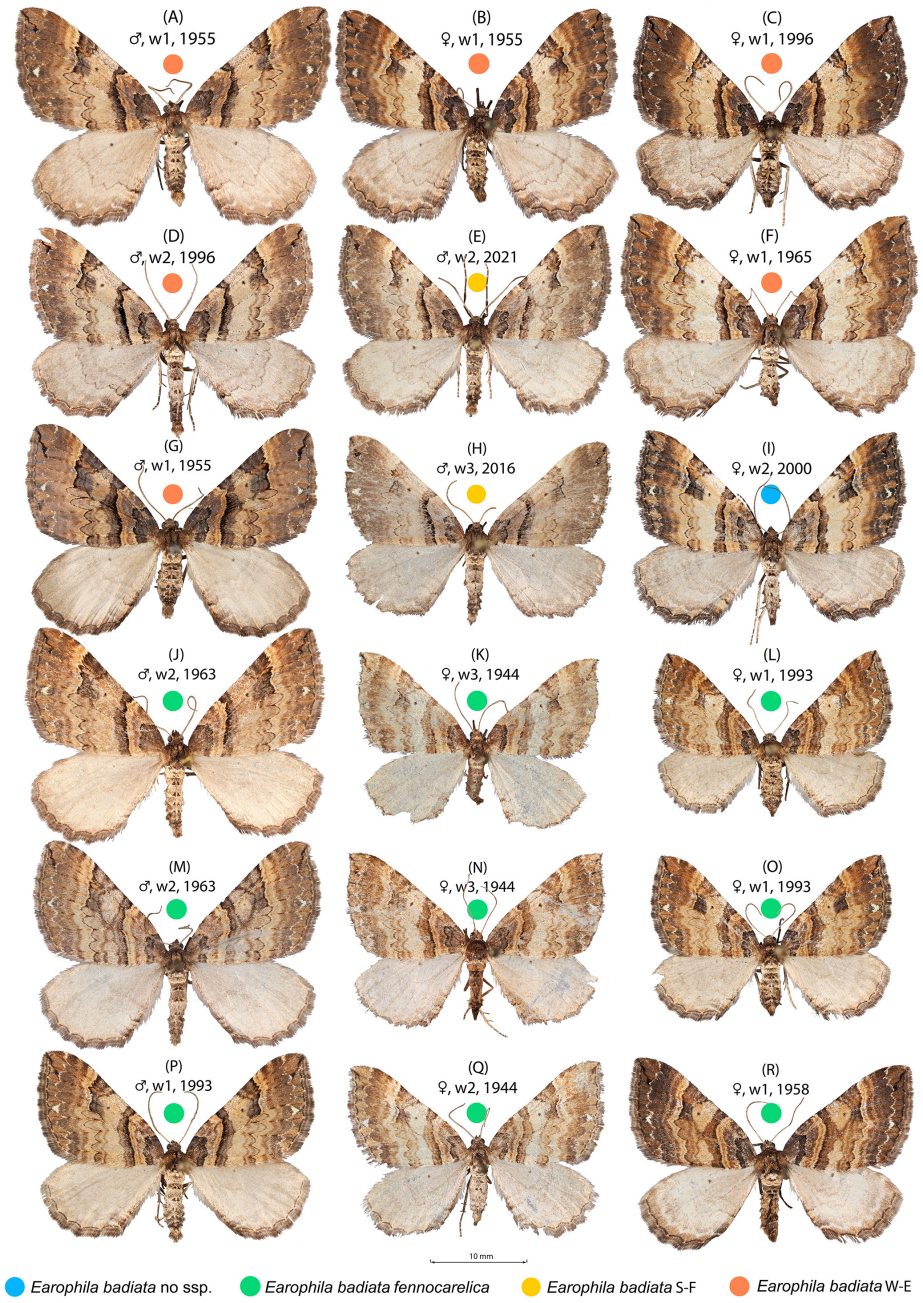
*Earophila badiata* adult specimens from different Eurasian populations. (A–H) *E. badiata badiata*, (J–R) *E. badiata fennokarelica*, and (I) *E. badiata* from northeastern China without subspecific affiliation. The sex, wear level (w1-3), and year of collection, in that order, are stated above each specimen. For more detailed information, see Table S1.

*Earophila badiata* is a univoltine nocturnal moth, adults flying in the spring months from March to early June throughout the distribution range. The larva is phytophagous on roses (*Rosa* spp.), and the pupa overwinters. In Finland, the *E. badiata fennokarelica* larva is referred (Seppänen, 1954; Mikkola, Jalas & Peltonen, 1985) to feed on *Rosa acicularis.* Both *E. badiata fennokarelica* and *R. acicularis* have a uniform distribution pattern in Finland covering only the province North Karelia in the eastern part of Central Finland (Lampinen, Lahti & Heikkinen, 2015). The late Finnish lepidopterist Veijo Mannelin in the 1960’s (Mikkola, Jalas & Peltonen, 1985, p. 129) and the first author (ME) in 1993–1995 reared *E. badiata* ex ovo originating from both the southwestern Finnish and northern Karelian populations on the same cultivated roses, the latter in Järvenpää, Finland located between the contemporary ranges of the species. Both noted an average difference in forewing colouration of the reared specimens (specimens in Figs. 2C, 2P, 2L) consistent with Kaisila’s description of the taxon *fennokarelica*. However, neither of the ex ovo series are large and complete enough for a statistical colour analysis.

Understanding species boundaries is essential for interpreting patterns of colonisation and hybridisation in contact zones. During our investigation of *E. badiata* populations in Finland and surrounding regions, we observed that morphological and genetic variation among specimens was inconsistent with the current species and subspecies delimitations. This highlighted the need for a thorough taxonomic reassessment of the *E. badiata* species group.

In this study, we present an integrative taxonomic revision of the Eurasian *E. badiata* species group, combining quantitative wing image analysis, comparative genitalia morphology, mitochondrial DNA barcoding (mtCOI), and micro-CT imaging. Differences in wing colouration and ornamentation patterns have long served as a quick and practical morphological tool for identifying lepidopteran species. Traditionally, such assessments have relied on visual inspection or “by-eye” comparison. However, despite their widespread use, these traits have rarely been quantified using objective image analysis methods—tools that are now common in evolutionary biology research but still underutilized in taxonomy. In the case of *E. badiata*, wing pattern variation has not been systematically assessed using quantitative approaches. In this study, we apply a quantitative wing image analysis methods similar to those previously used to explore the genetic-phenotypic associations and ecological and evolutionary significance of colour patterning in animals (Van Den Berg et al., 2020; Hemingson, Cowman & Bellwood, 2024; Nokelainen et al., 2024; Koch et al., 2025) adapting it here—novelly—to the taxonomic study of a nocturnal moth group.

We examined museum specimens from Eurasian populations of *E. badiata* to quantify forewing pattern variation and re-evaluated diagnostic genitalia morphology within this species group. To reconstruct phylogenetic relationships and assess haplotype diversity, we assembled a mtCOI dataset from BOLD Systems v.4 (http://www.boldsystems.org), including sequences from two *E. kolomietsi* paratypes and ten additional specimens from our morphological dataset. Finally, we combined these classical criteria with quantitative wing pattern analysis to assess their congruence and improve the resolution of species delimitation in this complex group.

## Materials and Methods

### Materials

We compiled three datasets (wing morphology, genitalia morphology, molecular mtCOI) to examine the morphology and molecular variation within the *E. badiata* species group. A set of 72 dried and pinned adult specimens from the collections of the Finnish Museum of Natural History, University of Helsinki (coll. FMHM, 53 specimens) and the private research collection of Mikael Englund (coll. Englund, 19 specimens) were selected for a closer examination (Tables 1, S1). The breakdown of our three datasets, based on the main variables of the forthcoming analyses, is shown in Table 1, and the full data from the morphological and molecular datasets are available in Tables S1 and S2.

**Table 1:** The breakdown of the three datasets of Palearctic *Earophila badiata* species group.

Dataset	Number of samples	Sex		Taxon affiliation						Wing wear		
		Male	Female	*Earophila badiata*					Earophila kolomietsi	1	2	3
				Badiata badiata	Badiata FI S	Fennokarelica	Tellensis	Not specified				
Wing morphology	72	40	32	23	6	41	0	2	0	28	37	7
Genital morphology	12	6	6	4	2	5	0	1	0	n/a	n/a	n/a
Molecular (mtCOI)	49	n/a	n/a	30	5	5	3	4	2	n/a	n/a	n/a

We chose specimens representing the subspecies *E. badiata badiata* and taxon *E. badiata fennokarelica* including four specimens from the type series of the latter taxon. Most of the specimens are from Finland or adjacent Russian Karelia, the geographical focus area of this study, but two are from Southern Sweden and one from Hungary, Russian Buryatia and North-Eastern China each. We annotated the specimens from the historical province of Karelia (FI Kar, RU Kar in Table S1) to the subspecific affiliation *fennokarelica,* while those from South-Western Finland (FI S-W), Sweden (SE), and Hungary (HU) we annotated a subspecific affiliation *badiata*. ‘We use “wear” to refer to the loss of scales, which may change the lepidopteran wing colour pattern, and “fading” to refer to the change of colour vividness during long term museum preservation. To control for wear and fading, we classified the wear of the specimens in three categories from little loss of scales (wear = 1) to severe loss of scales (wear = 3) and recorded the year of capture (Table S1, Fig. 2).

We chose a subset of 12 specimens from the wing morphology dataset for dissection to find and verify any diagnostic genitalia differences between the taxa *E. badiata badiata* and *E. badiata fennokarelica*, and the closely related species *E. kolomietsi* and *E. pseudobadiata*.

The specimens collected during 2000–2025 from the previously uninhabited areas in Southern Finland (FI S) we also affiliated to ssp. *badiata* based on our initial assessment of the wing colouration. The specimens from Russian Buryatia (RU Bu) and Nort-Eastern China (CH N-E) we left without a subspecific affiliation. A sample of 18 specimens included in the wing morphology data set representing different populations, sex, wear levels, and collection years is shown in Fig. 2.

To evaluate the intra- and interspecific DNA barcode differences within the *E. badiata* species group and prepare a haplotype analysis, we compiled a dataset of 49 mtCOI sequences. From these, 39 samples were retrieved from the BOLD database (Ratnasingham & Hebert, 2007) and the remaining 10 were newly sequenced in this study and uploaded to both BOLD (project code ‘BADIA’) and GenBank (accession numbers PV138762–PV138771). We classified the 35 barcode samples originating from Western Europe including Southwestern and Southern Finland to the subspecies *badiata*, those originating from the Finnish province of North Karelia (five samples) to the taxon *fennokarelica*. The three samples originating from Morocco were attributed to subspecies *tellensis*, and the remaining four *E. badiata* samples of Asian origin we left without a subspecific annotation. The two *E. kolomietsi* samples were sourced from paratypes collected in Kazakhstan (Tables 1, S2).

### Methods

#### Quantitative wing image analysis

We photographed all 72 specimens included in our wing morphology dataset using an Olympus OMD E-M1 mark II camera and Olympus Zuiko Digital ED 60 mm 1:2.8 Macro lens attached to a Kaiser repro stand and OM Capture v3.1 tethering software. Two dorso-laterally placed SmallRig P200 light panels set at 5600K were used as light sources, and colours were calibrated by using a XRITE ColorChecker Classic Mini card and a chart calibrated Human XYZ camera model in the micaToolbox for ImageJ (Schneider, Rasband & Eliceiri, 2012; Troscianko & Stevens, 2015). The camera white balance was also set at 5600K, image quality at ORF (Olympus raw format) and the lens aperture at F4. For each specimen, the right wing was selected by isolating the wing with a polygon and removing the surrounding background with the wand tool. A scalebar was drawn for each image and used to calculate the area, major length (length), and minor length (breadth) of the wings. To quantify and later compare the colouration of the moth wing images were converted to four channels, one for luminance (Lum) and three for colour, red-green opponent (X), blue-yellow opponent (Y), and saturation of the Euclidean distance of X and Y from 0 (Sat). Luminance was calculated by averaging the human X and Y cone-catch values, while the colour channels were constructed by converting the cone-catch to RNL chromaticity maps with a Weber fraction of 0.05.

Colour modelling with RNL chromaticity allowed us to quantify the average pixel value for the wing for each channel as well as the contrast and orientation of colour, not just luminance, patterns across six spatial scales (0.03125 cm, 0.0625 cm, 0.125 cm, 0.25 cm, 0.5 cm, one cm) with the largest scale being one cm using Gabor filters set to four different orientations (0°, 45°, 90°, 135°) (Barnett et al., 2017; Talas, Baddeley & Cuthill, 2017; Hancock, Cuthill & Troscianko, 2025). To ensure the orientation was the same for all wings, the wing images were rotated such that the longest dimension of the wing faced horizontally. For each scale, we measured the energy for each orientation, the average energy across all orientations, the verticality (vertical orientation–horizontal), and the directionality (max energy/mean energy across orientations). We also measured the sum for energy, verticality and directionality across all scales, as well as the scale where the energy was highest (maxEnergy) and the energy, verticality and directionality at that scale. This gave us 198 metrics (wing size and wing pattern) with which to compare differences in the appearance of our specimens.

To simplify these metrics, we used principal component analysis (PCA) to identify the features that most explain the variance. Linear mixed models (LMMs) were then used to determine which factors, if any (subspecies, sex, collection age, wear level, longitude and latitude) influenced the first three principal components (PC1, PC2, and PC3). We predicted that if the two subspecies are visually distinct, they would be significantly different in appearance and fall into discrete clusters. If a PC was found to be primarily explained by negative values, the PC was flipped (multiplied by -1) for clarity of language. The two specimens from East Asia without subspecific annotation were excluded from our LMMs. All numeric variables (latitude, longitude, collection age and wear level) were scaled to a mean of 0 and a standard deviation of 1. For each model, we used the collection group (Location + Year) as a random effect. For our analyses (LMMs and PCAs) we used R version 4.3.2 (R Core Team, 2023) with the lme4 package for our LMMs (Bates et al., 2015).

#### Genitalia morphology

To prepare the genitalia for photography and morphological comparisons, we detached abdomens from 12 (six males, six females) dried and pinned museum specimens originating from different *E. badiata* populations and exposed the genitalia following established preparation protocols (*e.g.*, Hardwick, 1950; Robinson, 1976; Sihvonen, 2001). The aedeagi were severed from the rest of the male genitalia, and the genitalia preparates were stained with Chorazol Black, dehydrated and stored in ethanol. For photography we placed each preparate to a comparable position in ethanol in a small glass bowl for photography through a Leica DM1000 LED stereo microscope with an attached Leica MC170 HD camera. We shot the photos of the signa through the bursa membrane, except for two specimens, where we cut the bursa open to get access to clearer view of the smaller signa. To evert the male vesica we incized the caecum near the tip, pushed the vesica towards the tip with a dog hair, and injected ethanol stained with Chorazol Black as described in more detail by Sihvonen (2001). The final genitalia photographs are mosaics of three to nine images each a stack of exposures from five to 17 planes. To stack the exposures, we used Zerene Stacker v1.04 software. To construct the mosaics and process the images, we used Adobe Photoshop v 25.4.0 and composed the final figures with Adobe Illustrator v28.1.

The micro-CT images appearing in the male exogenitalia figure are obtained by rendering micro-CT scans from a genitalia preparate (ME31) applying a modified protocol previously used to image dried undissected lepidopteran specimens (Englund et al., 2024). The stained male genitalia, fixed in ethanol, were air-dried and mounted on a paper strip using water-soluble glue before being scanned with a Nikon XT H 225 micro-CT scanner using a molybdenum target with 80 kV beam energy, 84 µA beam current, 1.42 s exposure time, 4,476 projections, and 2-frame averaging per projection. The total scan duration was approximately 3.5 h, resulting to a voxel size of 2.5 µm.

To enhance clarity, individual projection images were captured with 16-frame averaging while systematically adjusting the focus between shots to determine the optimal focus before initiating the full scan. We reconstructed the image with Nikon CT Pro 3D Version XT 6.9.1, while we conducted the segmentation and rendering in VGSTUDIO MAX 2024.3. Segmentation included digitally severing the genitalia from the paper support. The structures were then digitally coloured to approximate their natural appearance. Finally, we generated 3D footage, including both still images and videos to visualize the genitalia from multiple perspectives (Video S1).

#### Molecular methods

We extracted DNA from one to three leg tissues of dry collection specimens using the QIAamp DNA Micro Kit (Qiagen) for older specimens collected between 1990 and 2000, and the DNeasy Blood and Tissue kit (Qiagen) for more recent specimens collected between 2016 and 2021, following the manufacture’s protocols.

Amplification of the DNA barcode fragment (658 base pairs of the 5′ terminus) of the mitochondrial Cytochrome-C Oxidase I (mtCOI) gene was carried out using the universal primers LCO1490 and HCO2198 (Folmer et al., 1994) and MyTaq™ HS Red Mix (Bioline), with reaction volumes scaled down to 12.5 µl. For samples that failed to amplify the full-length barcode, a smaller COI fragmentation (∼440 bp) was amplified using primers C1-J-1718 (Simon et al., 1994) and HCO2198. The PCR program for both primer pairs began with an initial denaturation at 95 °C for 5 mins, followed by 40 cycles of 96 °C for 30 s, 50 °C for 30 s, and 72 °C for 90 s, with a final extension at 72 °C for 10 mins. All lab procedures were conducted at the Luomus DNA Laboratory, Helsinki. Sanger sequencing was outsourced to the Institute for Molecular Medicine Finland (FIMM), Helsinki, using an ABI3730xl platform.

Sequence alignment for the mtCOI gene was performed using the MUSCLE algorithm, as implemented in MEGA11 (Tamura, Stecher & Kumar, 2021), which was also used to calculate pairwise genetic distances using the Kimura 2-parameter (K2P) model, both within and between species. Three samples of the sister subspecies *E. badiata tellensis* were used as outgroup for the phylogenetic analysis. GenBank accession numbers or BIN numbers from BOLD Systems for the sequences used are presented in Table S2.

Phylogenetic analysis was conducted using maximum likelihood (ML) with IQ-TREE v.2.1.3 (Minh et al., 2020). The best partitioning scheme was determined using ModelFinder (Kalyaanamoorthy et al., 2017), selected based on the corrected Akaike Information Criteria. To ensure a comprehensive tree search, 100 independent ML searches were performed, and the best-scoring tree was selected for further analysis. Branch support was then assessed using the ultrafast bootstrap approximation method (UFBoot2) with 1,000 replicates, incorporating nearest-neighbour interchange optimisation (-bnni) (Minh, Nguyen & Haeseler, 2013). The best-fitting substitution model was HKY+F+I. The resulting tree was visualized and rooted in FigTree v.1.4.3 (Rambaut, 2016) and further modified in Adobe Illustrator CS6 or CorelDRAW v24. Haplotype relationships were assessed using a median-joining haplotype network created with PopArt v.1.7.2 (Leigh & Bryant, 2015).

## Results

### Quantitative wing image analysis

The first three principal components (PC1-3) explained 51% of the variance in our colour and wing size measures. PC1 (28% of the variance) can be interpreted to mainly correspond to colour patterning (saturation and blue-yellow contrast) at intermediate scales (0.0625 cm and 0.125 cm), PC2 (12%) to mean and contrast luminance, and PC3 (11%) to luminance and colour patterning at large scales (one cm) orientated 135 degrees. See the Data S1 for principal component loadings.

In our linear regressions, PC1 and PC2 were most strongly and significantly affected by sex, followed by wear, while subspecies had no significant effect on these principal components (Table 2). Males were significantly darker (lower PC2) and less contrasting in colour (lower PC1) than females. However, we found only weak support (in PC3) for the diagnostic difference in forewing colour vividness and contrast between *E. badiata badiata* and *E. badiata fennokarelica* reported in literature (Hausmann & Viidalepp, 2012; Kaisila, 1945; Mikkola, Jalas & Peltonen, 1985).

**Table 2:** Linear mixed model estimates and statistics.

Principal component	Fixed effect	Degrees of freedom	Estimate	SE	*t*value	*p*value
PC1	Sex = male	63	−1.100	0.167	−6.570	<0.001
	Subspecies = f	17	0.655	0.476	1.377	0.186
	Specimen_wear	30	−0.389	0.099	−3.941	<0.001
	Specimen_age	4	−0.276	0.093	−2.965	0.038
	Latitude	29	0.069	0.150	0.458	0.650
	Longitude	20	−0.349	0.744	−0.470	0.644
PC2	Sex = male	62	−0.605	0.169	−3.575	0.001
	Subspecies = f	28	−0.280	0.511	−0.548	0.588
	Specimen_wear	52	0.419	0.103	4.068	0.000
	Specimen_age	12	−0.368	0.106	−3.471	0.005
	Latitude	39	−0.209	0.158	−1.323	0.193
	Longitude	33	2.130	0.793	2.686	0.011
PC3	Sex = male	63	−0.284	0.230	−1.233	0.222
	Subspecies = f	63	−1.316	0.646	−2.037	0.046
	Specimen_wear	63	0.058	0.134	0.428	0.670
	Specimen_age	63	0.356	0.124	2.863	0.006
	Latitude	63	0.468	0.204	2.292	0.025
	Longitude	63	−0.128	1.011	−0.126	0.900

**Figure 3:**
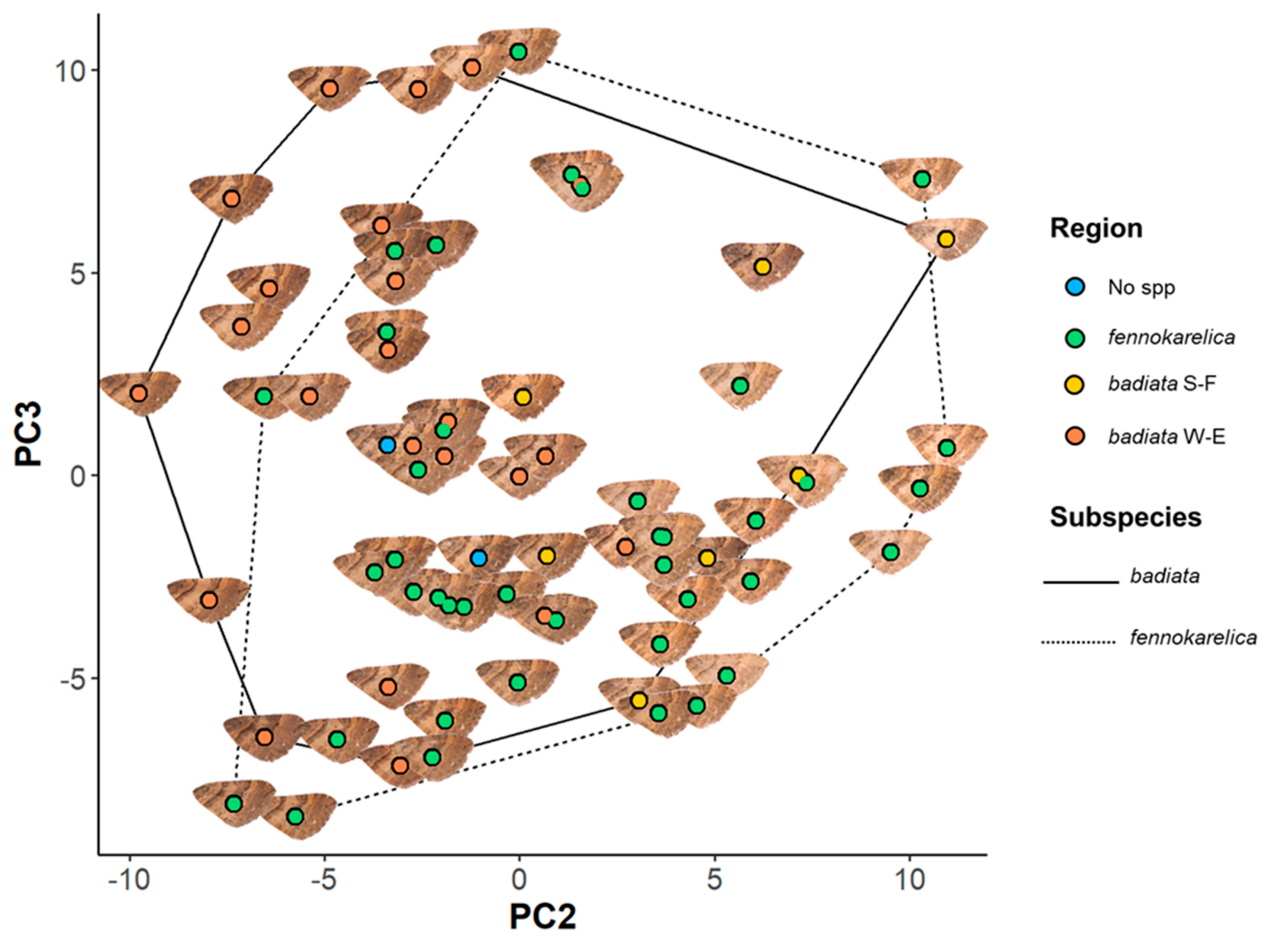
Forewing colouration principal component analysis (PCA) mapping. The coloured circles and wing images represent the 72 specimens in the wing morphology dataset plotted on a plane with PC2 being the horizontal axis and PC3 the vertical axis. The colour of the circle indicates the regional origin of each specimen. For the full data of the specimens, see Table S2 and for the associated ID numbers see Fig. S3.4.

The only other factor that had a significant effect on PC1 and PC2 was the specimen age, which had the inverse effect on PC2 compared to wear. For PC3, subspecies affiliation did have a large but only weakly significant effect, with *E. badiata fennokarelica* showing lower luminance and colour contrast on average at larger spatial scales than *E. badiata badiata,* although there was considerable overlap between the two (Fig. 3). The longitude of specimen origin increased PC2 and latitude PC3, the specimens of southwestern origin positioning more often in the upper left quadrate and the specimens with northeastern origin in the lower right quadrate. The forewing colour pattern of the specimens colonising South Finland appear to fall between those of the specimens from North Karelia (ssp. *fennokarelica*) and South-West Finland (ssp. *badiata*).

There was, however, a high degree of overlap between the PC values for specimens annotated as E. *badiata badiata* and *E. badiata fennokarelica* (Fig. 3.) and we could not infer any practical rules how to delimit the ssp. *badiata* and ssp. *fennokarelica* based on forewing colour pattern.

**Figure 4:**
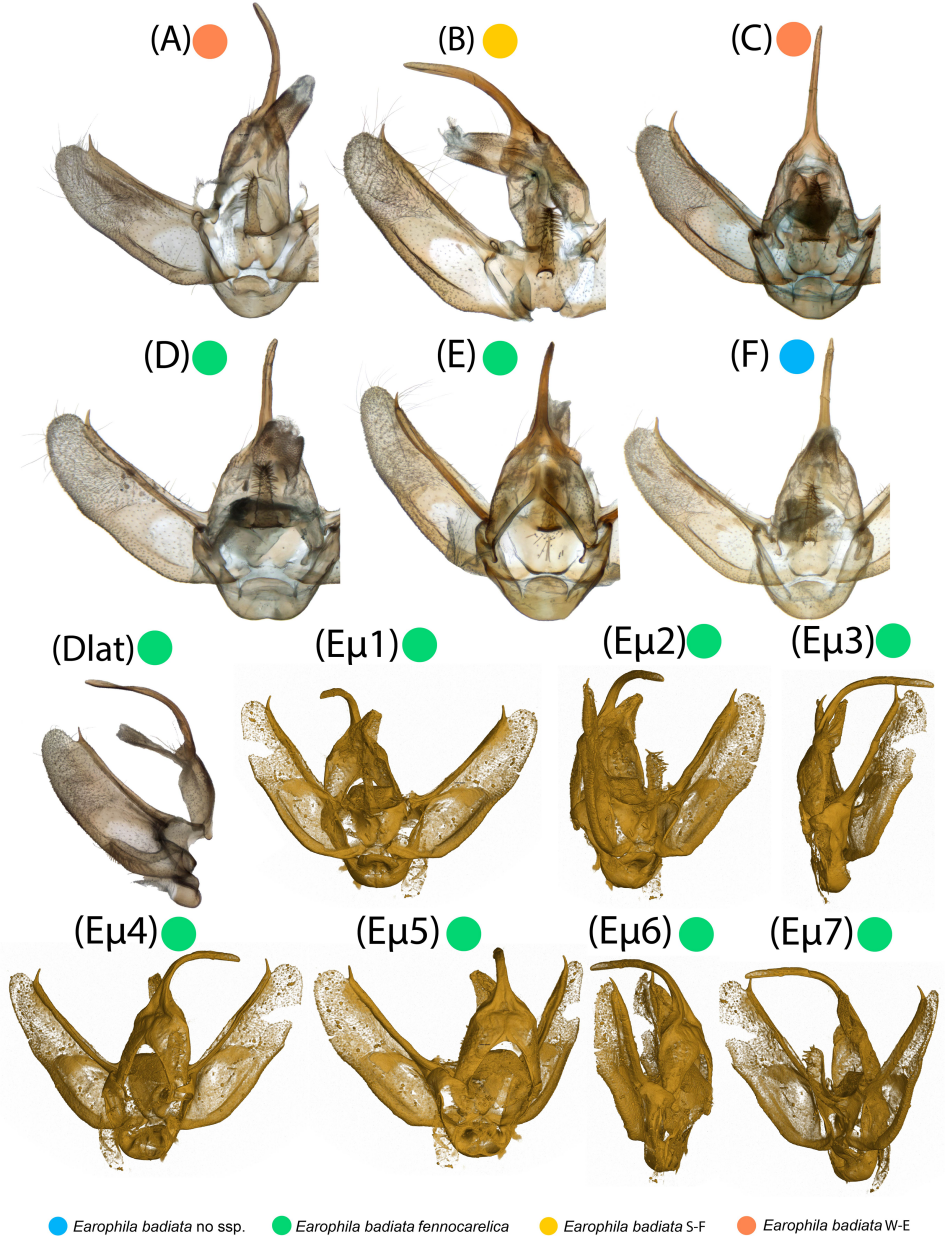
Male exogenitalia. Microscopic photos in caudal view: (A–C) *E. badiata badiata*, (D–E) *E. badiata fennokarelica*, (F) *E. badiata* (Russia, Buryatia). Microscopic photograph in lateral view: (Dlat) *E. badiata fennokarelica*. (Eµ1-7) Micro-CT images of *E. badiata fennokarelica* from different view angles. A video footage rendered from the same micro-CT scan is available in Fig. S4.

### Genitalia morphology

Microscopic photographs and micro-computed tomography (micro-CT) images of exogenitalia of the six dissected male specimens are portrayed in Fig. 4. The exogenitalia exhibit some individual variation, and variation attributable to deformation of the structures during natural dehydration of the specimens, and slightly different angles of photography. We could identify no diagnostic differences in genitalia morphology between specimens originating from different geographical areas or subspecies.

**Figure 5:**
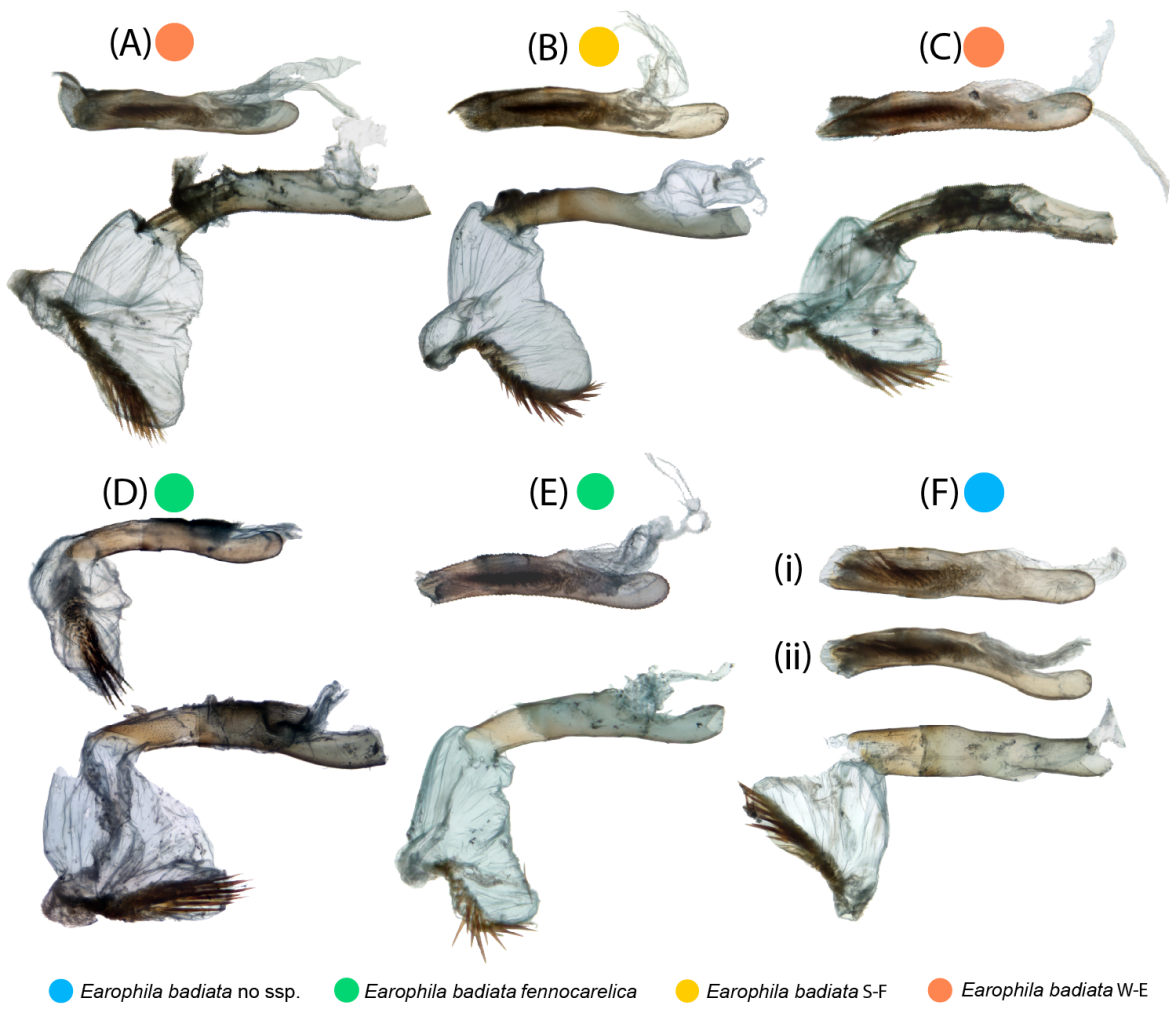
Male endogenitalia. Microscopic photos; aedeagi unmanipulated (above) and aedeagi caeci cut and vesicae everted (below): (A–C) *E. badiata badiata*, (D–E) *E. badiata fennokarelica*, (F) *E. badiata* (Russia’ Buryatia), (Fi) ventral view, (Fii) lateral view.

Microscopic photographs of the endogenitalia (aedeagus and vesica) of the six male specimens are portrayed in Fig. 5. In one specimen (Fig. 5D), the vesica was naturally partly everted, and in two specimens (Figs. 5D, 5F), the vesica was ruptured, resulting in incomplete eversion. The variation particularly in the shape of everted vesicae is attributable to the same sources as with the exogenitalia described above. A slightly flattened tubular aedeagus can appear in varying shapes portrayed from slightly different view angles in stacked two-dimensional photos (Figs. 5Fi, 5Fii).

**Figure 6:**
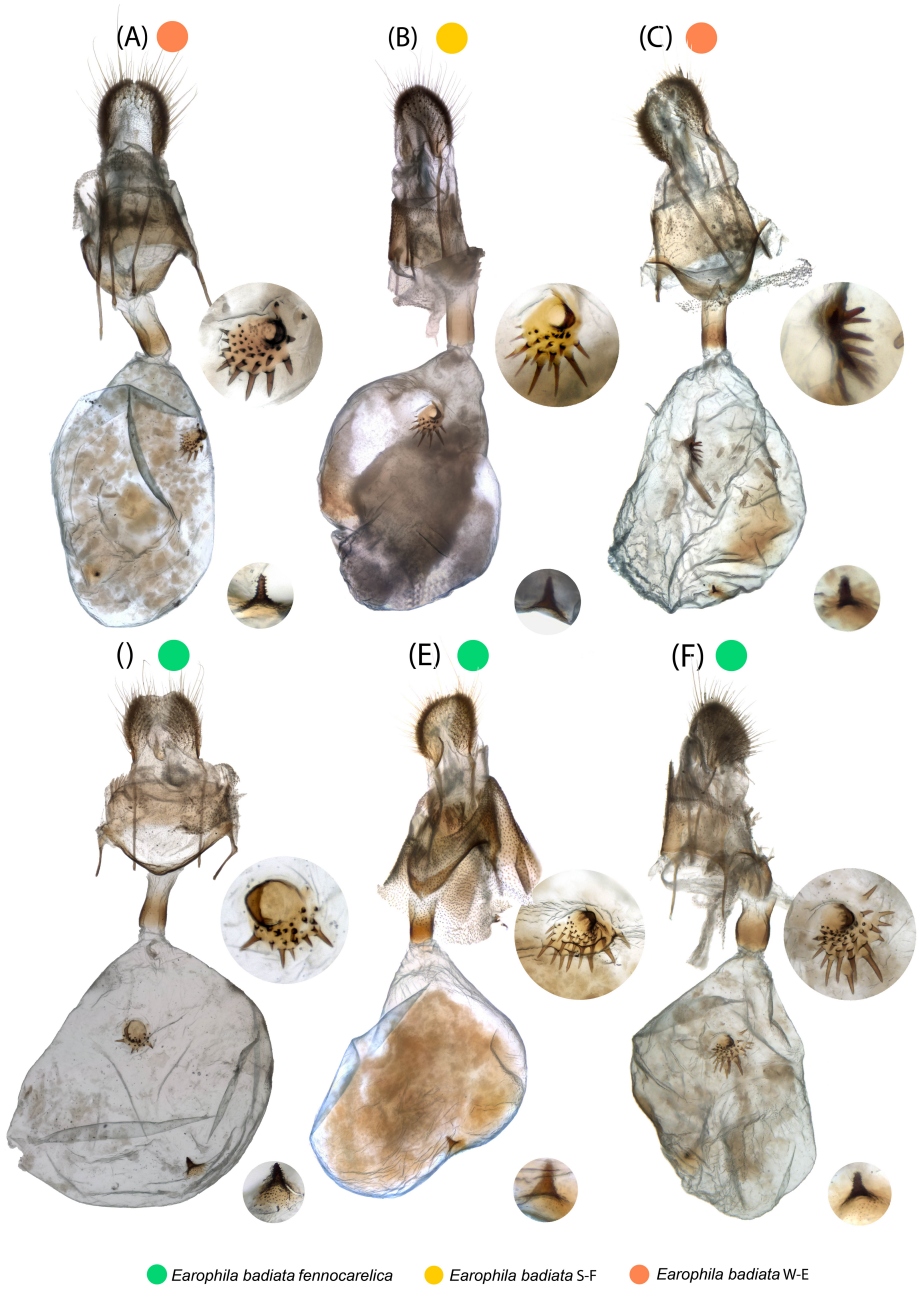
Female genitalia. Microscopic photos of the entire genitalia apparatus; (A–C) *E. badiata badiata*, (D–F) *E. badiata fennokarelica*. The two signa inside the bursa copulatrix are magnified in the circles to the right of each panel.

The varying opacity of the bursae content and membrane, and the penetration of staining agent cause variance in the clarity of the photos shot through the membrane. In particular, spermatophores in varying state of decomposition may result in different transparency of the bursa copulatrix. The female genitalia are uniform in shape, except the corpus bursae. This membranous structure is loosely elongated, or sac-like in shape, but we consider this shape variation to fall within intraspecific variation or being attributable to artefacts arising from differences in deformation during natural dehydration and/or sample position. There are two notable signa with spines of varying sizes and shapes attached inside the membrane of bursa copulatrix (Fig. 6). The larger stellate signum protrudes outwards from the bursa membrane and the smaller triangular signum correspondingly inwards. These are located uniformly in same locations in different specimens: stellate signum in posterior part and triangular signum in anterior part.

We also carefully evaluated the diagnostic differences between the genitalia morphology of the three species presented in the drawings and key in the original description of *E. pseudobadiata* by Vasilenko (2007).

### Molecular analysis

**Figure 7:**
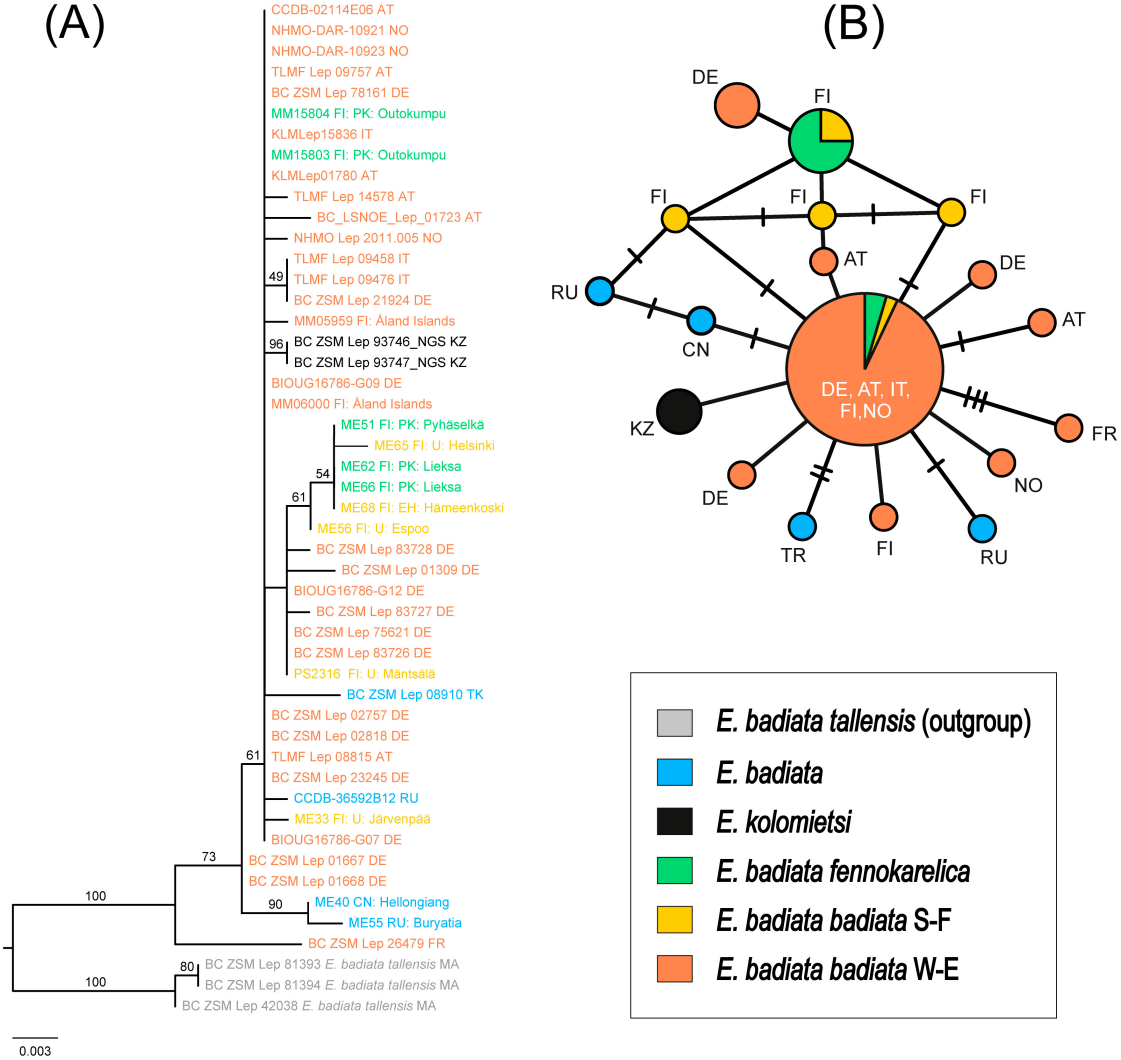
Phylogeny and haplotypes. (A) Maximum likelihood (ML) tree inferred from IQ-TREE based on mtCOI sequences within the *Earophila badiata* species group, with three outgroups from *E. badiata tellensis*. (B) The median-joining network of mtCOI haplotypes. Colours correspond to the species and subspecies shown in ML tree. The size of circles represents the number of samples included in each haplotype, with larger circles indicating a higher number of samples. Small black lines between haplotypes indicate mutations, with each branch representing a single base pair change.

The ML tree supported the *E. badiata* species group as monophyletic, with 100% ultrafast bootstrap support (Fig. 7A). The three samples of *E. (Anticlea) badiata tellensis* from Morocco were clearly distinct from the *E. badiata* species lineage and served as outgroups in the tree. Within this lineage, *E. badiata, E. badiata fennokarelica*, and *E. kolomietsi* were intermixed. Pairwise genetic distances among the ingroup taxa ranged from 0.12% (*E. badiata badiata*–*E. kolomietsi*) to 0.63% (*E. badiata badiata*–*E. badiata fennokarelica*), with 2.41% to 2.98% divergence from the outgroups.

The haplotype network revealed a total of 18 distinct haplotypes among 46 individuals, with 14 haplotypes unique to a single sample (Fig. 7B). The network displayed a star-like topology, with most haplotypes connected to the central haplotype. This central haplotype was the most frequent and widely distributed across Europe, shared by individuals of *E. badiata badiata* (from both South Finland S-F and West Europe W-E) and *E. badiata fennokarelica*. Another shared haplotype was identified between *E. badiata badiata* (S-F) and *E. badiata fennokarelica*. The haplotype of *E. kolomietsi* differed by a single base pair from the central haplotype. Haplotypes of *E. badiata* from Türkiye, China, and Russia were also closely related to the central haplotype, differing by up to four base pairs.

## Discussion

The subspecies rank has been applied under fundamentally different taxonomic concepts since at least 1844 (Burbrink et al., 2022), a debate that is closely linked to the broader and long-standing “species problem” in biology (*e.g.*, Mayr, 1996; De Quieroz, 2007; Reydon & Kunz, 2021, to mention a few). At a minimum, taxonomic entities are expected to be distinguishable from one another in some consistent and replicable way—whether through morphology, genetics, behaviour, or other lines of evidence. However, cases such as cryptic species illustrate that these differences may not always be obvious or detectable without detailed analyses. To evaluate this in the *E. badiata* species group, we conducted an integrative analysis combining quantitative wing colour assessment, comparative genitalia morphology, and molecular phylogenetics, including haplotype analysis of the mitochondrial COI marker. Our goal was to test the validity of diagnostic traits previously proposed in the literature for separating the described Palearctic taxa within this group.

Our forewing quantitative image analysis, novel in a taxonomic context, revealed previously unreported sexual dimorphism in *E. badiata*, potentially reflecting different selective pressures or ecological roles between males and females (Cueva Del Castillo, Gonzales-Zertuche & Ramirez-Delgado, 2021; Binns et al., 2025). The clear detection of sex-based differences also supports the method’s sensitivity and suitability for identifying biologically meaningful variation. We could also verify that both scale loss and ageing during long-term museum preservation alter markedly lepidopteran wing colours, which arise as a product of light reflectance in the pigments and nanostructures in scales covering the surface of lepidopteran wings (Thayer & Patel, 2023; Stavenga, 2024).

Mikkola, Jalas & Peltonen (1985, p. 84) presented doubts about the validity of the subspecific rank of taxon *fennokarelica* pertaining to the poor condition of the type specimens and quality of the photos in Kaisila’s original description of the taxon *fennokarelica* (Kaisila, 1945). Based on our quantitative image analysis, we could not reaffirm the diagnostic differences in forewing colouration between the subspecific taxa *E. badiata badiata* and *E. badiata fennokarelica* presented by Kaisila (1945). A cline or phenotypic plasticity driven by a thermal and continental climatic gradient from south-west to north-east in Finland, rather than a genetic dichotomy caused by reproductive isolation justifying a subspecific taxonomic separation, could explain the results of our quantitative wing image analysis (Fig. 3, Data S1). We can resolve this issue by abstaining from the common interpretation (Hausmann & Viidalepp, 2012; Mikkola, Jalas & Peltonen, 1985; Scoble, 1999) of the “forma geographica” being equivalent to “subspecies” but treat it as a morphological form of *E. badiata* without a taxonomic trinomial rank, as presented in the original description.

Functional and comparative genitalia morphology is also considered to be a valuable tool to diagnose insect species since according to the lock-and-key theory incompatibilities in male and female genitalia are rapidly evolving and cause reproductive isolation (*e.g.*, Masly, 2012; Mikkola, 2008; Omiya et al., 2024). However, the evidence to support the lock-and-key hypothesis remains debated (*e.g.*, Eberhard & Ramirez, 2004; Mutanen, 2006). Especially older descriptions of genitalia are often based on hand-drawn illustrations and verbal descriptions, which may be prone to subjective interpretations by both the authors and readers of the description. Other issues such as inherent individual variation, variation in sample preparation, positioning for photography, and absorption of staining agent can also render genitalia morphology less dependable in some cases. Based on our comparative genitalia morphology assessment (Figs. 4–6), we agree with Kaisila’s statement that there are no noticeable differences in the genitalia morphology between *E. badiata badiata* and *E. badiata fennokarelica* (Kaisila, 1945). We also agree with the opinion presented by Hausmann & Viidalepp (2012, p. 179) about “the absence of significant differential feature[s]” between *E. badiata, E. kolomietsi*, and *E. pseudobadiata*, as all the diagnostic differences between these three taxa pertaining to details in genitalia morphology presented by Vasilenko (2007) are explainable by infraspecific variation, artefacts arising from positioning and imaging the preparates, or variation in deformation of the relevant structures during dehydration. Consequently, we consider *E. kolomietsi* and *E. pseudobadiata* to be junior synonyms of *E. badiata* until further evidence is presented to delimit these taxa from *E. badiata*.

All 46 Eurasian samples in our mtCOI molecular dataset, including two type specimens *E. kolomietsi,* show convincing monophyly and low intraspecific variance (<0.62%) whereas the three Moroccan *E. badiata tellensis* samples form a separate cluster in our molecular phylogeny (Fig. 7A) with a noticeable genetic distance (>2.4%) from the rest of the dataset. The haplotype network (Fig. 7B) corroborates these results, showing a star-like structure centred on a dominant haplotype shared by *E. badiata badiata* and *E. badiata fennokarelica*, with *E. kolomietsi* differing by only a single mutation. Such pattern often reflects recent population expansion, shallow divergence, or the result of extensive gene flow across populations (Avise, 2009; Kivelä et al., 2025). However, the low intraspecific variance of the mtCOI gene in our dataset does not exclude the possibility of marked variance in other genetic markers among the different populations (Joshi et al., 2024), and the anecdotal evidence from ex ovo rearing experiments would support that possibility. The current sympatry and likely interbreeding between the Finnish populations would render any new attempts to show such differences quite complicated and prone to identification problems.

Allopatry and genetic distance between *E. badiata tellensis* and the Eurasian *E. badiata* merit a further examination of the relation between these taxa.

We encourage wider application of quantitative wing image analyses in integrative insect taxonomy to establish objective and quantifiable morphology-based species delimitation criteria and recommend micro-CT imaging rendered to mimic the natural coloration of sclerotized structures to better understand the three-dimensional morphology of insect genitalia. A more detailed analysis is warranted to reveal the exact mechanisms underlying sexual dimorphism and wing pattern variation in *E. badiata*.

## Conclusions

We expected the specific taxa *E. kolomietsi, E. pseudobadiata* (Vasilenko, 2003; Vasilenko, 2007)*,* and the subspecific taxon *E. badiata fennokarelica* (Kaisila, 1945) to be separable from each other and the nominate form of *E. badiata badiata* (H_1_) using the diagnostic morphological traits presented in the original descriptions of these taxa. Based on our analyses of three datasets comprising samples from 111 specimens representing the taxa under revision, we could not confirm the presented interspecific diagnostic differences between these taxa. Moreover, we found little genetic variance in the barcode region (mtCOI) in our molecular dataset supporting the results from our comparative morphological analysis of forewing colour patterns and genitalia. Consequently, we conclude that there is just one Eurasian specific taxon within the *E. badiata* species group (H_0_), and all other described taxa within that species group should be considered junior synonyms of *E. badiata*.

Based on our findings, we propose the following taxonomic changes:

1. *Earophila kolomietsi* Vasilenko, 2003 is classified to synonymy *with E. badiata* syn. n.
2. *Earophila pseudobadiata* Vasilenko, 2007 is classified to synonymy with *E. badiata* syn. n.
3. *Earophila badiata* ssp. *fennokarelica* Kaisila, 1945 is classified to synonymy with *E. badiata* syn. n.

We discovered a previously unreported sexual dimorphism of *E. badiata* and could verify that specimens wear and aging during long-term museum do change lepidopteran wing appearance.

## Supplemental Information

10.7717/peerj.20620/supp-1 Supplemental Information 1 Morphology Dataset

10.7717/peerj.20620/supp-2 Supplemental Information 2 Molecular Dataset

10.7717/peerj.20620/supp-3 Supplemental Information 3 Image Analysis Supplement

10.7717/peerj.20620/supp-4 Supplemental Information 4 Male Genitalia 3D Footage

10.7717/peerj.20620/supp-5 Supplemental Information 5 Earophila badiata new mtCOI sequences DNA sequences: PV138762–PV138771..

## Additional Information and Declarations

### Data Availability

The following information was supplied regarding data availability: The data is available in the Supplemental Files. The data is available at Zenodo: Englund, M., Hancock, G., Laiho, E., Mappes, J., Sihvonen, P., Söderholm, M. S., Turunen, A., & Lee, K. M. (2025). Supplemental Files for the manuscript: Quantitative image analysis applied to revise the taxonomy of the Palearctic *Earophila badiata* species group (Lepidoptera: Geometridae: Larentiinae) (Data set). Zenodo. https://doi.org/10.5281/zenodo.17017663
The data is available at MorphoSource:
https://www.morphosource.org/projects/000780291?locale=en
Project ID: 000780291 micro-CT scan file ID: 000780296
https://doi.org/10.17602/M2/M780296
#D footage ID: 000780300
https://doi.org/10.17602/M2/M780300
Image Analysis Data & Scripts are available at Github and Zenodo: https://github.com/GeorgeHancock471/Badiata-Image-Analysis
George RA Hancock. (2025). GeorgeHancock471/Badiata-Image-Analysis: Badiata-Image-Analysis (v1.0). Zenodo. https://doi.org/10.5281/zenodo.17788174
The MtCOI/DNA barcode sequences are available at BOLD: - https://portal.boldsystems.org/recordset/DS-BADIA25 (project code ‘BADIA’). - DOI: doi.org/10.5883/DS-BADIA25. The raw sequence data are available at NCBI BioProject: PV138762–PV138771.
