# Supplementary material for: Quantitative image analysis applied to revise the taxonomy of the Palearctic Earophila badiata species group (Lepidoptera: Geometridae: Larentiinae)
Source: PeerJ. 2026 Feb 19;14:e20620. doi: 10.7717/peerj.20620 (PMC12925412; doi:10.7717/peerj.20620)
Supplement: Supplemental Information 3 [file peerj-14-20620-s003.docx]

*Earophila badiata* species group revision: Supplementary information S3

Quantitative Image Analysis Supplement

Image Setup


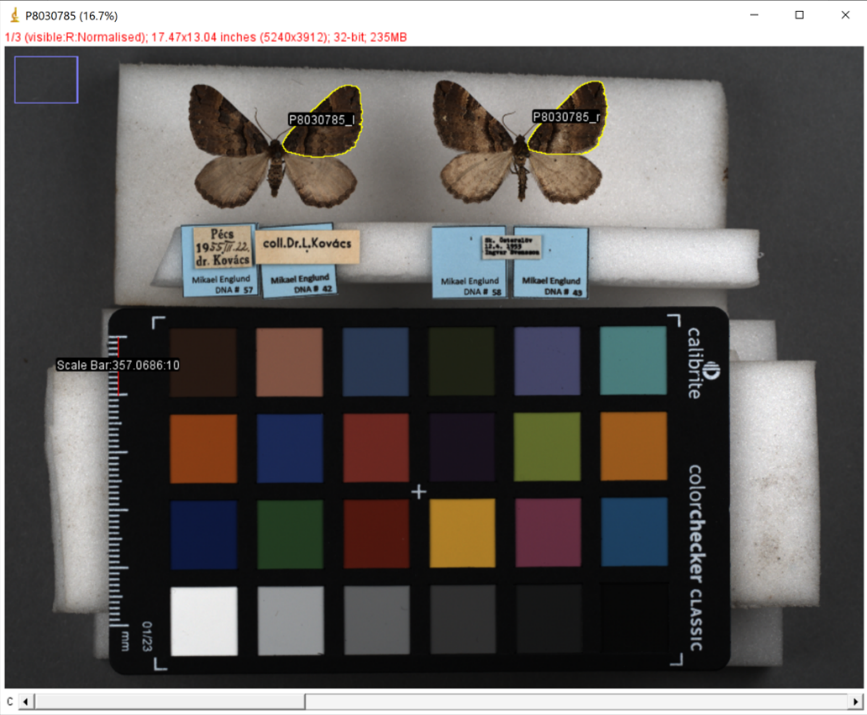


Figure S3.1. Example image setup. Two moths are arranged with their photo label on the left and right, along with a luminance standard. The right wing for each moth is labelled with a region of interest (ROI). The ROIs, shown in yellow, are labelled using the photo number and whether the moth is on the left (l) or the right (r). The scalebar shown in red is marked for 10mm. It denotes the number of pixels, in this case 357, per mm.

Masking Method


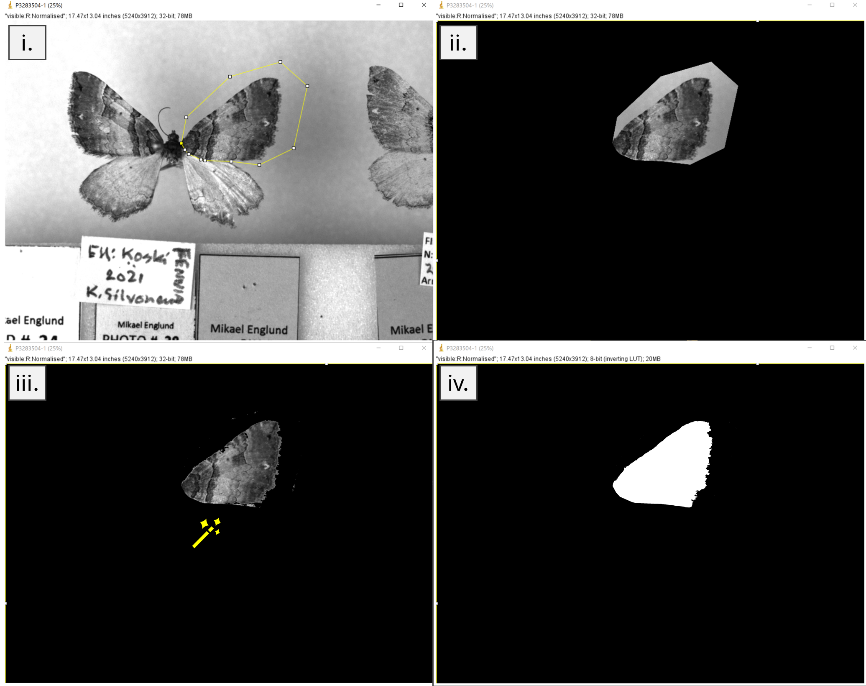


Figure S3.2. Example wing masking. i) A loose polygon is drawn around the wing. ii) The area surrounding the polygon is masked out in black. iii) The remainder of the background is masked using the wand tool. iv) The wing is converted to a white selection and any gaps are filled in white.

Gabor Filtering


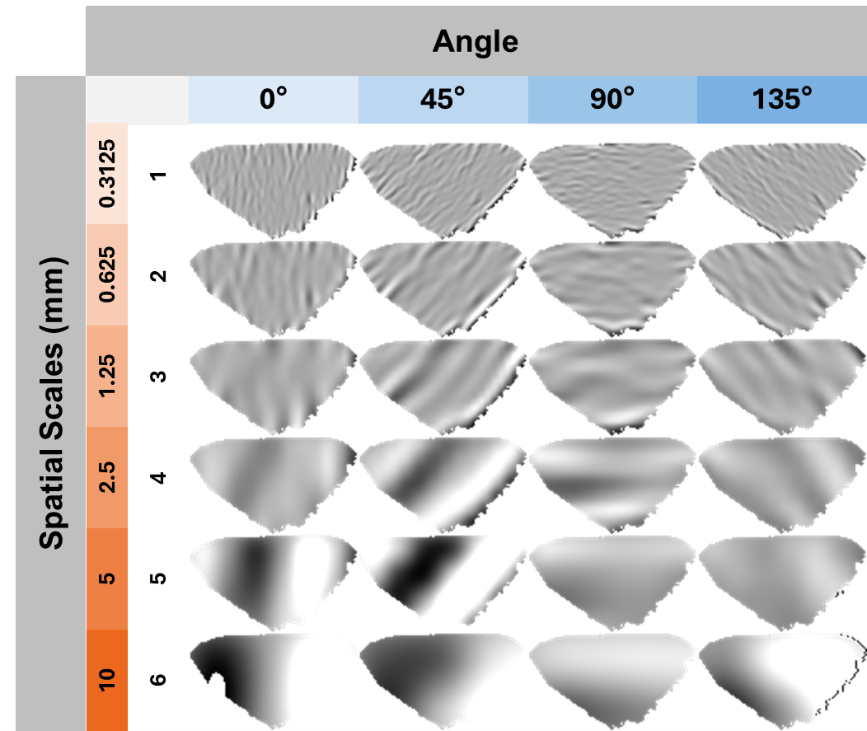


Figure S3.3. Example Gabor filtering of the moth wing for the luminance channel. The x-axis shows the orientation of the Gabor filter in degrees, and the y-axis shows the spatial scale in mm and the associated number code for that scale (1-6). Larger spatial scales correspond with larger patterning. Images show the contrast at that given scale.

Principal Component Analysis

Table S3.1. Contributions to principal components (PC), PC1, PC2, and PC3 shown in ranked absolute order for variables that contributed greater than or equal to 0.10. NB for PC1 and PC2 the values shown have been flipped (multiplied by -1) as the majority were negative.

| Variable | PC1 | Variable | PC2 | Variable | PC3 |
| --- | --- | --- | --- | --- | --- |
| Sat_contrastAvg_2 | 0.125992 | Lum_StdDev | 0.172167 | Lum_contrast_6_135 | 0.172347 |
| Y_contrastAvg_2 | 0.125414 | Lum_Mean | 0.16637 | Lum_SumEnergy | 0.16873 |
| Sat_contrastAvg_3 | 0.123298 | Lum_contrastAvg_1 | 0.164732 | Lum_contrastAvg_6 | 0.168691 |
| Y_contrastAvg_3 | 0.122069 | Lum_contrast_2_45 | 0.161346 | Lum_MaxPower | 0.166406 |
| Sat_contrastAvg_4 | 0.120101 | Lum_contrast_1_0 | 0.158437 | Sat_contrast_6_135 | 0.161151 |
| Y_contrastAvg_4 | 0.119426 | Lum_contrast_3_0 | 0.157817 | Lum_MaxFreq | 0.156466 |
| Sat_contrast_4_45 | 0.11697 | Lum_contrastAvg_2 | 0.157292 | Y_contrast_6_135 | 0.154954 |
| Sat_contrast_1_90 | 0.116805 | Lum_contrast_2_0 | 0.156094 | Sat_MaxFreq | 0.14951 |
| Sat_contrastAvg_1 | 0.116417 | Lum_contrast_1_135 | 0.155317 | X_contrast_6_45 | 0.148898 |
| Y_contrastAvg_5 | 0.116074 | Lum_contrast_1_45 | 0.151001 | Sat_contrastAvg_6 | 0.148774 |
| Sat_contrast_2_90 | 0.115881 | Lum_contrastAvg_3 | 0.150114 | Sat_SumEnergy | 0.148739 |
| Y_contrast_4_45 | 0.115856 | Lum_contrastAvg_4 | 0.149814 | Y_SumEnergy | 0.143592 |
| Y_contrastAvg_1 | 0.115786 | Lum_contrast_4_135 | 0.143267 | Y_contrastAvg_6 | 0.143572 |
| Sat_contrast_1_135 | 0.115482 | Lum_contrast_2_135 | 0.142806 | X_MaxPower | 0.140044 |
| Sat_StdDev | 0.11521 | Lum_contrast_3_45 | 0.141148 | X_SumEnergy | 0.138821 |
| Sat_contrastAvg_5 | 0.114433 | Lum_contrast_1_90 | 0.140256 | X_contrastAvg_6 | 0.138729 |
| Y_contrast_2_90 | 0.114256 | Lum_contrast_4_90 | 0.136207 | Sat_MaxPower | 0.137705 |
| Y_contrast_1_135 | 0.11391 | X_Mean | -0.13614 | Lum_contrast_6_0 | 0.137474 |
| Y_contrast_1_90 | 0.112942 | Lum_contrast_4_0 | 0.135978 | Y_directionality_1 | -0.13343 |
| Y_contrast_5_45 | 0.112785 | Lum_contrastAvg_5 | 0.134699 | Y_MaxPower | 0.132583 |
| Y_StdDev | 0.112359 | Lum_contrast_3_135 | 0.132435 | Sat_directionality_1 | -0.13121 |
| Y_contrast_3_45 | 0.111697 | Sat_Mean | -0.12588 | Y_MaxFreq | 0.126598 |
| Sat_contrast_5_45 | 0.111591 | Major_mm | -0.12483 | Lum_contrast_6_45 | 0.125917 |
| Sat_contrast_3_45 | 0.111474 | Minor_mm | -0.12278 | Y_contrast_6_90 | 0.122825 |
| X_contrast_1_90 | 0.11085 | Y_Mean | 0.122679 | X_MaxFreq | 0.121197 |
| Sat_contrast_3_90 | 0.109762 | Lum_contrast_4_45 | 0.120194 | Sat_contrast_6_90 | 0.116823 |
| Y_contrast_2_0 | 0.109004 | Lum_contrast_2_90 | 0.112477 | Y_verticality_1 | 0.116427 |
| X_contrastAvg_2 | 0.108696 | Lum_contrast_3_90 | 0.110532 | Lum_contrast_6_90 | 0.115656 |
| Y_contrast_3_90 | 0.107632 | Lum_contrast_5_90 | 0.10894 | Y_directionality_2 | -0.11298 |
| Sat_contrast_2_0 | 0.107422 | Lum_contrast_5_135 | 0.105875 | Sat_verticality_1 | 0.112913 |
| X_contrastAvg_4 | 0.107292 |  |  | Sat_directionality_2 | -0.11058 |
| Y_contrast_4_0 | 0.10694 |  |  | Sat_contrast_6_45 | 0.10354 |
| Sat_contrast_4_0 | 0.106795 |  |  | X_verticality_1 | 0.103069 |
| Sat_contrast_3_135 | 0.104417 |  |  | Y_contrast_6_45 | 0.102039 |
| X_contrastAvg_3 | 0.103462 |  |  | Sat_contrast_6_0 | 0.101823 |
| Y_contrast_4_90 | 0.103192 |  |  | X_contrast_6_0 | 0.100715 |
| Y_contrast_3_135 | 0.10309 |  |  | Y_verticality_2 | 0.100701 |
| Sat_contrast_3_0 | 0.103043 |  |  |  |  |
| X_contrastAvg_1 | 0.102545 |  |  |  |  |
| Y_contrast_3_0 | 0.10217 |  |  |  |  |
| Sat_contrast_4_90 | 0.101714 |  |  |  |  |
| Sat_contrast_2_135 | 0.100508 |  |  |  |  |
| X_contrast_2_90 | 0.10003 |  |  |  |  |

Principal Component Plots


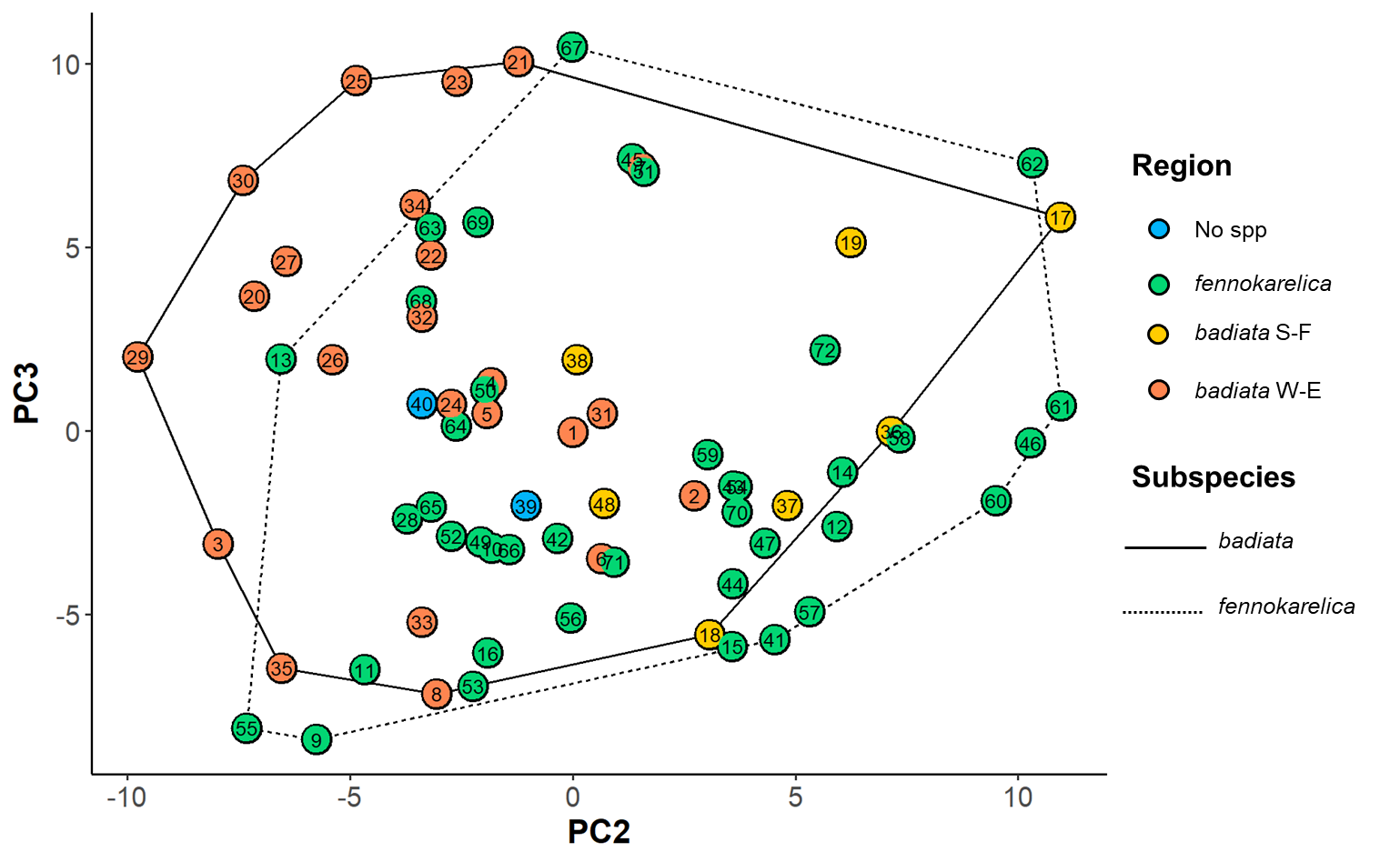


Figure S3.4. Forewing colouration PCA mapping for subspecies. The coloured circles represent the 72 specimens in the wing morphology dataset plotted on a plane with PC2 being the horizontal axis and PC3 the vertical axis. The colour of the circle indicates the regional origin of each specimen and the number inside the circle is the identification number for each specimen. For the full data of the specimens, see Table S2.


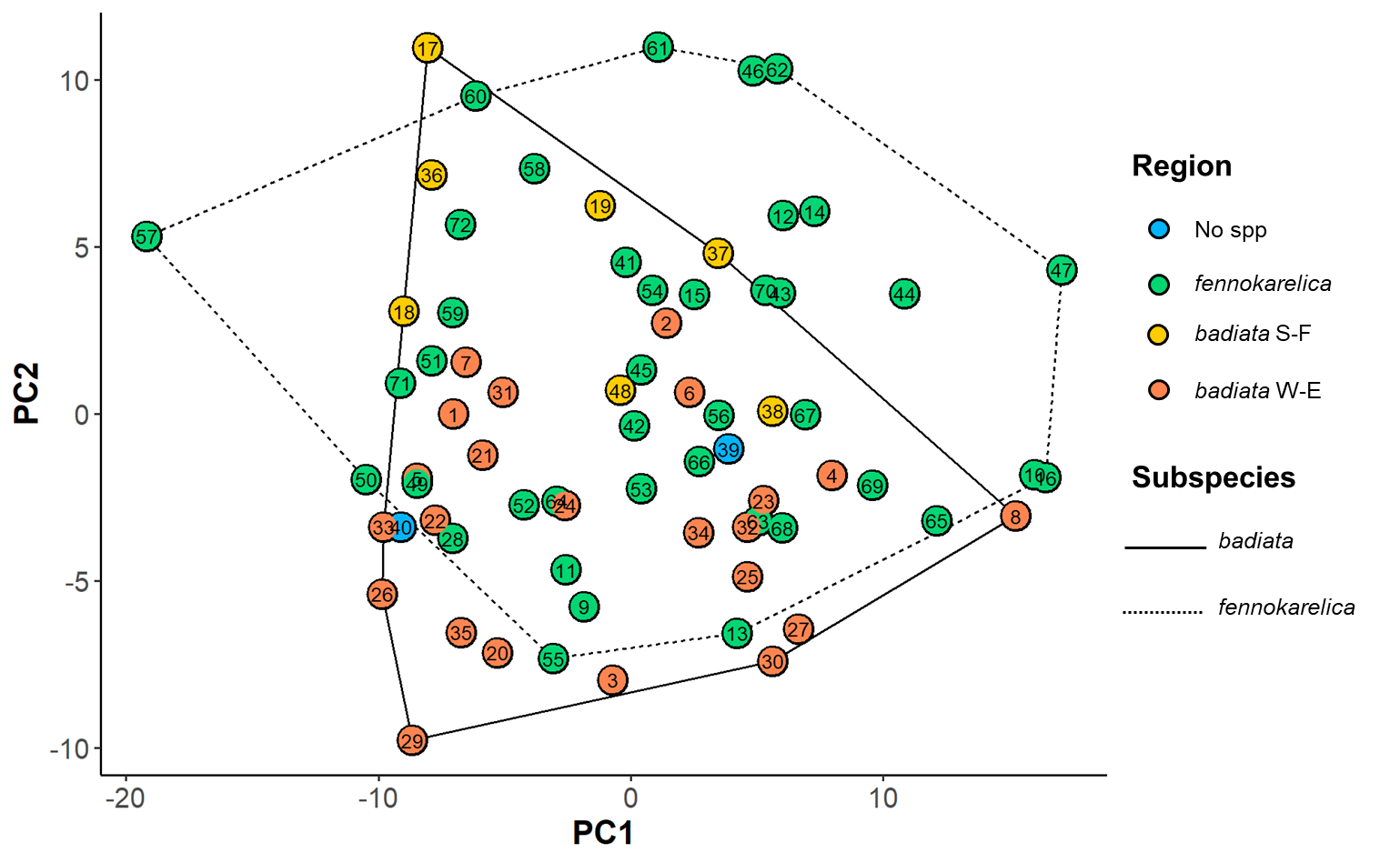


Figure S3.5. Forewing colouration PCA mapping for subspecies. The coloured circles represent the 72 specimens in the wing morphology dataset plotted on a plane with PC1 being the horizontal axis and PC2 the vertical axis. The colour of the circle indicates the regional origin of each specimen and the number inside the circle is the identification number for each specimen. For the full data of the specimens, see Table S2.


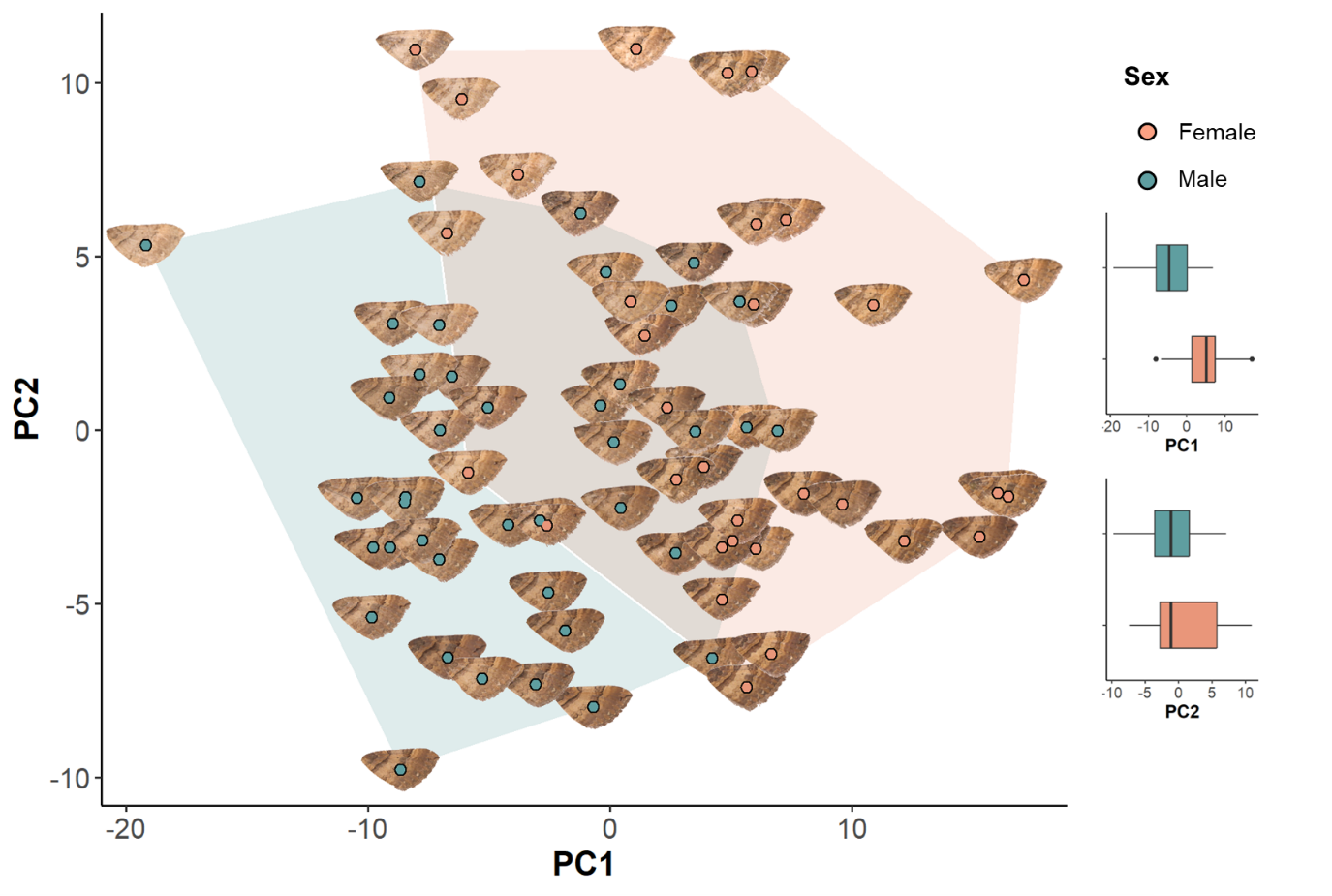


Figure S3.6. Forewing colouration PCA mapping for sex. The coloured circles and wing images represent the 72 specimens in the wing morphology dataset plotted on a plane with PC1 being the horizontal axis and PC2 the vertical axis. The colour of the circles and boxplots indicates the sex of each specimen. The right-hand boxplots show individual comparisons for PC1 and PC2 between sexes.
